# Strain-Level Diversity of Secondary Metabolism in *Streptomyces albus*

**DOI:** 10.1371/journal.pone.0116457

**Published:** 2015-01-30

**Authors:** Ryan F. Seipke

**Affiliations:** School of Molecular and Cellular Biology, University of Leeds, Leeds, LS2 9JT, United Kingdom; University of Strathclyde, UNITED KINGDOM

## Abstract

*Streptomyces* spp. are robust producers of medicinally-, industrially- and agriculturally-important small molecules. Increased resistance to antibacterial agents and the lack of new antibiotics in the pipeline have led to a renaissance in natural product discovery. This endeavor has benefited from inexpensive high quality DNA sequencing technology, which has generated more than 140 genome sequences for taxonomic type strains and environmental *Streptomyces* spp. isolates. Many of the sequenced streptomycetes belong to the same species. For instance, *Streptomyces albus* has been isolated from diverse environmental niches and seven strains have been sequenced, consequently this species has been sequenced more than any other streptomycete, allowing valuable analyses of strain-level diversity in secondary metabolism. Bioinformatics analyses identified a total of 48 unique biosynthetic gene clusters harboured by *Streptomyces albus* strains. Eighteen of these gene clusters specify the core secondary metabolome of the species. Fourteen of the gene clusters are contained by one or more strain and are considered auxiliary, while 16 of the gene clusters encode the production of putative strain-specific secondary metabolites. Analysis of *Streptomyces albus* strains suggests that each strain of a *Streptomyces* species likely harbours at least one strain-specific biosynthetic gene cluster. Importantly, this implies that deep sequencing of a species will not exhaust gene cluster diversity and will continue to yield novelty.

## Introduction

More than two-thirds of all therapeutic small molecules used in medicine are derived or inspired from complex natural products produced by filamentous actinobacteria, most notably *Streptomyces* spp. [1]. *Streptomyces* spp. are predominantly known as filamentous soil bacteria that have a differentiating mycelial life-cycle, which begins with spore germination and outgrowth of a vegetative mycelium and ends with production of reproductive aerial hyphae and the formation of unigenomic spores [2]. Aerial hyphae production and sporulation is often accompanied by the production of secondary metabolites. These secondary metabolites are most likely used to outcompete neighbouring organisms [3]. Biotechnology has exploited many of these natural products as anticancer, antiviral, insecticidal, herbicidal, antibacterial, antifungal and immunosuppressive compounds [4].

Growing global concerns about resistance to antibacterial agents has led to a renaissance in bioprospecting and natural product discovery. The resurgence of interest in natural products is greatly aided by the relatively inexpensive cost to sequence genomes of strains that produce promising bioactive small molecules. One-hundred and forty-two streptomycete genomes are available in DDBJ/EMBL/Genbank. This dataset has made it abundantly clear that *Streptomyces* spp. only express a mere fraction of their biosynthetic genes under standard laboratory growth conditions. Activation of silent biosynthetic gene clusters and characterisation of their products represents a major potential source for new lead compounds for industry and is an area in which synthetic biology holds huge promise [5].

In order to capitalise on available genomic resources, systematic analyses of secondary metabolism are required. Doroghazi and Metcalf provided the first comparative analysis of secondary metabolism in organisms with closed genomes from the phylum *Actinobacteria*, which included eight *Streptomyces* species and revealed, for good reason, why this taxa has been the focus of rigorous genomic and biochemical analyses over the years [6]. Recently, Ziemert et al. performed a focused analysis of the secondary metabolism in 75 sequenced *Salinispora* species identified a total of 124 biosynthetic pathways encoded by the genus and provided insight into population-level genetic exchange of biosynthetic pathways in marine environments [7]. Doroghazi et al. recently developed a method for classification of gene clusters into families and used this approach to analyse the biosynthetic potential of 830 sequenced Actinobacteria, which they found to contain a total of 11,422 gene clusters comprising 4,122 gene cluster families [8]. More analyses of these type will be required in order to drive the fields of natural product discovery and synthetic biology forward and maximise the promise held by genome mining actinomycetes.

*Streptomyces albus*, which is one of the most widely geographically distributed streptomycetes and has been isolated from diverse environments including sponges, sea sediments and insects [9–14]. The archetype member of this species is *S. albus* J1074 which is a derivative of *S. albus* G in which the *salI* restriction system was deleted to better enable transformation [15]. *S. albus* J1074 has therefore been used as a host for heterologous expression of several natural product gene clusters, including cyclooctatin [16], fredericamycin [17], iso-migrastatin [18], moenomycin [19], napyradiomycin [20], steffimycin [21] and thiocoraline [22] and there has recently been renewed interest in further developing this expression platform because of its fastidious growth and naturally minimised genome [23]. The clear ability of *S. albus* J1074 to heterologously biosynthesise diverse and important natural products suggests strains of *S. albus* may encode important natural product gene clusters of their own, a question which genomics and genome mining is only just now beginning to address. As more researchers sequence closely related strains it makes necessary an understanding of strain-level diversity in secondary metabolism. With this view in mind, here I report a strain-level analysis of secondary metabolism for six sequenced *S. albus* strains. A total of 48 biosynthetic gene clusters were identified and approximately 18 specify the core secondary metabolome of *S. albus*, 14 are auxiliary gene clusters and 16 are strain-specific, indicating there is still appreciable chemical diversity to be discovered at the strain level.

## Results and Discussion

### A multilocus phylogeny of *Streptomyces* spp. reveals significant redundancy in sequenced organisms

Many of the 142 genome sequences available for *Streptomyces* spp. originate from so-called environmental isolates and their taxonomic classification remains enigmatic. A multilocus phylogeny was reconstructed in order to infer a taxonomic relationship among sequenced *Streptomyces* spp. and assess redundancy in the genomic database. Multiple loci were used to infer phylogenetic relationships because of well recognised problems with the use of solely the 16S rRNA gene as a phylogenetic marker, as it only provides an accurate and reliable classification to the genus level of streptomycetes [24] likely due to extensive recombination in the evolutionary past [25]. The loci selected for this study were those employed by previous multilocus phylogenies of streptomycetes: 16S rDNA, *aptD* (ATP synthase), *gyrA* (DNA gyrase subunit A), *recA* (recombination protein), *rpoB* (RNA pol subunit) and *trpB* (tryptophan biosynthesis) [26,27]. 16S rDNA sequences could not be identified in some draft genome sequences. This is presumably a result of an inadequacy with DNA assembly software to process the multiple copies (five to seven copies) of the ribosomal RNA locus streptomycetes are known to harbour. The partial 16S rDNA sequences (variable region IV) that were retrieved had a maximum pairwise divergence of ∼5% over 292 nt (determined by blast analysis). With the motivation to include as many genome sequences in this analysis as possible, the decision was therefore made to exclude the 16S rRNA gene as a phylogenetic marker for this study. Partial DNA sequences for *atpD, gyrA, recA, rpoB* and *trpB*, corresponding to regions targeted by well established oligonucleotide primer sequences employed in phylogenetic analyses [26,27] were retrieved from Genbank (see methods). Due to the poor quality of some of the genome sequences and/or the absence of some of these genes entirely, ∼14% (20 genomes) were excluded from this analysis. Redundant genomes for type-strains were also excluded, namely *S. bottropensis* ATCC 25435 ([Genbank:AOCF00000000]), *S. clavuligerus* ATCC 27064 ([Genbank:ADGD00000000]) and *S. albus* J1074 ([Genbank:ABYC00000000]).

**Figure 1:**
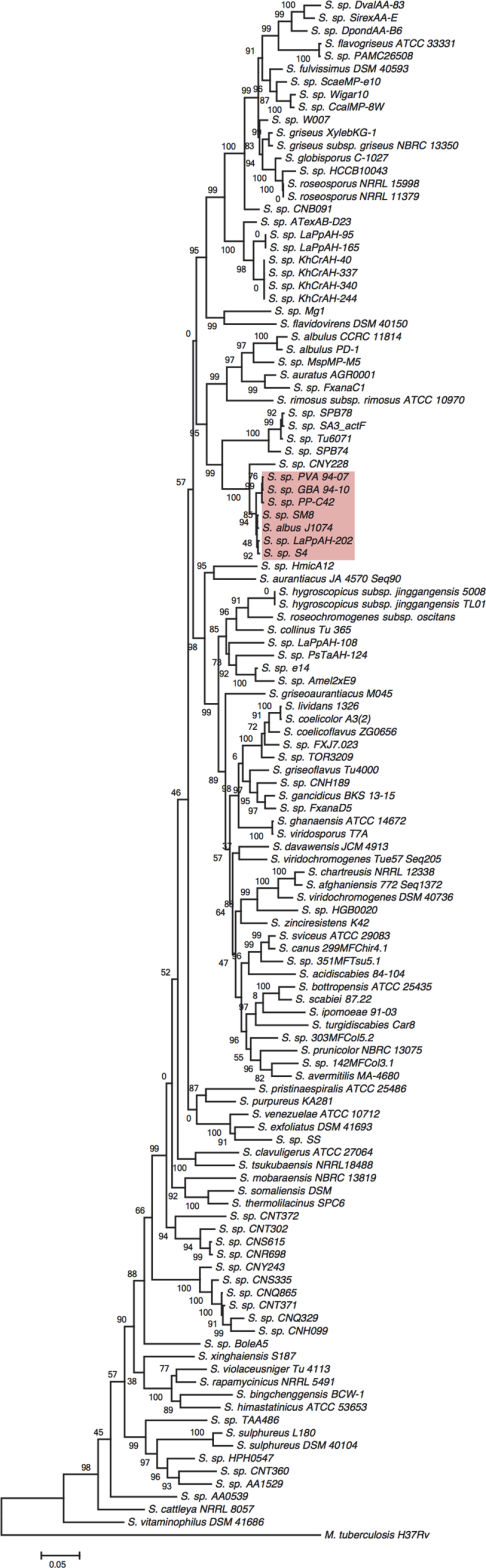
An approximately maximum likelihood phylogenetic tree of sequenced *Streptomyces* species. A phylogeny was inferred for *Mycobacterium tuberculosis* and 120 sequenced streptomycetes based on concatenated partial sequences of *aptD-gyrB-recA-rpoB*. FastTree local support values (expressed as a whole number) are indicated at each node. *Streptomyces albus* strains are highlighted in red. The scale bar indicates 5% estimated sequence divergence.

An approximately maximum-likelihood phylogenetic tree based on concatenated *aptD-gyrB-recA-rpoB-trpB* gene fragments (2566 nt in total) was constructed (Fig. 1). Overall, there was good separation and statistical support for most of the branches in the tree. Interestingly, the tree suggested that many *Streptomyces* species have been sequenced more than once. To further analyse this, the concatenated *aptD-gyrB-recA-rpoB-trpB* gene fragments were next binned into operational taxonomic units (OTUs) with a shared identity threshold of 97%, which is a widely used threshold for species-level classification [28]. Approximately 70% (82 out of 120) of the sequenced streptomycetes analysed here correspond to a unique species of *Streptomyces* (S1 Table). The most (over-)represented species for which a genome sequence is available is *Streptomyces albus* (seven sequences in total). The availability of multiple genome sequences for a single species enables valuable analyses of the diversity and distribution of secondary metabolism which have only now become possible and will help inform and direct bioprospecting efforts in *Streptomyces* spp.

### Secondary metabolism in *S. albus*

The archetype member of the *S. albus* clade is *S. albus* J1074 [15] commonly used as a heterologous expression host [16–22]. The six additionally sequenced strains of *S. albus* were identified more recently and their isolation was motivated, at least in part, by bioprospecting in unexploited microbial niches and include: *S*. sp. PVA-94-07, *S*. sp. GBA 94-10, *S*. sp. SM8, *S*. sp. PP-C42, *S*. sp. LaPpAH-202 and *S*. sp. S4. Details of *S. albus* strains are summarised in Table 1. The poor quality of the genome sequence available for *S*. sp. PP-C42 (>7,000 contigs) prevented its inclusion in this analysis so therefore a total of six *S. albus* genomes were analysed here.

**Table 1:** Accessions and genomic features of *Streptomyces* *albus* strains.

**Organism**	**Accession**	**Genome size (Mb)**	**Number of contigs**	**Source**	**Reference**
*S. albus* J1074	NC_020990	6.83	Closed	Unknown	[23]
*S*. sp. PVA-94-07	ASHE00000000	7.10	20	Nordic fjords	[9]
*S*. sp. GBA 94-10	ASHF00000000	7.22	34	Nordic fjords	[9]
*S*. sp. PP-C42	AEWS00000000	6.46	7,074	Baltic Sea sediment	[11]
*S*. sp. SM8	AMPN00000000	7.15	513	North Sea sponges	[10]
*S*. sp. LaPpAH-202	ARDM00000000	7.00	36	Plant-ants	[14]
*S*. sp. S4	CADY00000000	7.61	269	Leaf-cutting ants	[12]

Gene clusters encoding putative secondary metabolites were identified using antiSMASH 2.0 [29] and, crucially, were edited to best reflect published experimental data. Three independent analyses of secondary metabolism in *S. albus* J1074 have been conducted in this year [9,23,30]. These analyses disagree with regard to the total number of putative biosynthetic gene clusters encoded by *S. albus* J1074. Briefly, these analyses were hindered by using the draft version of the *S. albus* J1074 genome sequence [30], use of an earlier version of antiSMASH [23] and not taking into consideration experimental data [9,23].

*S. albus* strains encode between 25–30 biosynthetic gene clusters with *S. albus* J1074 encoding the least (25) and *S*. sp. PVA-94-07 encoding the most gene clusters (30) (Table 2). A pairwise comparison of gene clusters revealed significant redundancy in the putative secondary metabolites produced by *S. albus* strains. Importantly, the pairwise comparison also revealed that between 3 and 21% of gene clusters harboured by an individual strain are in fact strain-specific (Table 2), which suggests that gene cluster diversity may not be exhausted by deep-sequencing multiple strains of a single species, a prediction that was recently validated for the marine actinomycete, *Salinispora* spp. [7].

**Table 2:** Pairwise comparison of gene clusters encoding putative secondary metabolites from *Streptomyces albus* strains.

	***S. albus* J1074**	***S*. sp. PVA-94-07**	***S*. sp. GBA 94-10**	***S*. sp. SM8**	***S*. sp. LaPpAH-202**	***S*. sp. S4**	**No. unique gene clusters**
*S. albus* J1074	25 (100%)	20 (80%)	19 (76%)	20 (80%)	23 (92%)	21 (84%)	2 (8%)
*S*. sp. PVA-94-07		30 (100%)	28 (93%)	20 (66%)	21 (70%)	21 (70%)	1 (3%)
*S*. sp. GBA 94-10			29 (100%)	19 (65%)	20 (68%)	20 (68%)	1 (3%)
*S*. sp. SM8				26 (100%)	21 (80%)	21 (80%)	3 (11%)
*S*. sp. LaPpAH-202					27 (100%)	22 (81%)	3 (11%)
*S*. sp. S4						28 (100%)	6 (21%)

The percentage in braces reflects the total number of gene clusters conserved in the pairwise comparison with respect to the strains listed vertically.

### The core secondary metabolome of the *S. albus* clade

*S. albus* strains encode the production of a conserved set of 18 secondary metabolites (Table 3). Eight of these metabolites are produced by most (if not all) streptomycetes and include: desferrioxamine and aerobactin-like siderophores [31], ectoine osmolytes [32], hopanoid membrane components [33], carotenoid pigments [34], tetrahydroxynapthalenes [35], the morphologically-important lantipeptide, SapB [36] and volatile geosmins whose function is still obscure nearly 50 years after its discovery [37]. The remaining 10 gene clusters harboured by all *S. albus* strains are not as widely conserved at the genus level. These metabolites include: candicidin, a polyene antifungal compound [12,38], the respiratory chain inhibitor and anti-anti-apoptotic agent, antimycin [39,40], an antibacterial, similar to gramicidin [38], the volatile terpenoid antibacterial, albaflavenone [41], and the antifungal alteramide [30]. Interestingly, alteramide was first isolated from a sponge-associated *Alteromonas* spp. [42] and its production by *S. albus* J1074 was only observed after engineering its expression and is the first report of alteramide biosynthesis in the genus *Streptomyces* [30]. Additionally, the core secondary metabolome of *S. albus* includes five unknown products encoded by NRPS (2), Type I PKS (1) and bacteriocin (2) gene clusters (Table 3).

**Table 3:** The core secondary metabolome of *Streptomyces albus*.

**Predicted biosynthetic system**	**Putative product**	***S. albus* J1074**	***S*. sp. PVA-94-07**	***S*. sp. GBA 94-10**	***S*. sp. SM8**	***S*. sp. LaPpAH-202**	***S*. sp. S4**
Hopene / Squalene synthase	Hopanoids	308626..335220	647179..673767	608447..635035	Multiple contigs***	NZ_KB890705.1 522286..548874	CADY01000122.1 571..33079
NRPS-independent siderophore synthase	Desferrioxamine	4740450..4752270	4783002..4794822	4778566..4790386	NZ_AMPN01000107.1 27451..38705	NZ_KB890704.1 497713..509533	CADY01000052.1 71864..83684
NRPS-independent siderophore synthase	Aerobactin-like	1268164..1283196	1461319..1476318	1419952..1434951	Multiple contigs***	NZ_KB890708.199818..114849	CADY01000147.1 1..13852
Ectoine synthase	Ectoine	5635346..5645744	5646629..5657027	5645248..5655646	NZ_AMPN01000262.1 1748..15421	NZ_KB890727.1 65721..76119	CADY01000033.1 3246..13644
Phytoene / polyprenyl synthetase	Carotenoids	6401161..6430221	6435309..6461258	6426764..6452713	NZ_AMPN01000036.1 9070..37323	NZ_KB890733.1 196543..229868	CADY01000098.1 59202..91885
Terpene synthase	Geosmin	1531759..1554059	1713992..1736292	1672644..1694944	NZ_AMPN01000357.1 6256..26511	NZ_KB890732.1 367671..389971	CADY01000157.1 6106..28406
Terpene	Albaflavenone	1865241..1887220	2122786..2144075	2082541..2103830	NZ_AMPN01000386.1 17700..38989	NZ_KB890732.1 22700..43989	CADY01000162.1 47721..69010
Type III PKS	Tetrahydroxynapthalene	6520374..6561471	6555445..6596542	6545260..6586357	NZ_AMPN01000015.1 1..12125	NZ_KB890733.1 61315..102412	CADY01000095.1 47527..88624
Type I PKS	Candicidin	6566408..6721648	6586760..6741995	6576564..6731817	Multiple contigs***	Multiple contigs***	Multiple contigs***
Type I PKS*	Unknown	6776680..6838639	6797026..6858963	6786847..6849240	Multiple contigs***	NZ_KB890710.1 1..60636	Multiple contigs***
Hybrid NRPS / PKS*	Antimycin	6730563..6755198	6750919..6775544	6740741..6765365	NZ_AMPN01000430.1 1.7480	Mutiple contigs***	CADY01000091.1 16873..41495
Hybrid NRPS / PKS	Alteramide	224752..275005	560646..610065	522971..572390	NZ_AMPN01000189.1 1..20256	NZ_KB890705.1 438436..488689	CADY01000120.1 1..40866
Hybrid NRPS / PKS**	Unknown	6755272..6776675	6775619..6797021	6765440..6786842	Multiple contigs***	NZ_KB890710.1 60641..82030	CADY01000091.1 1..16795
NRPS	Gramicidin-like	3877105..3982798	3970595..4076299	3946534..4052238	Multiple contigs***	Multiple contigs***	CADY01000205.1 1..90613
NRPS	Unknown	4469477..4514441	4546088..4590455	4542814..4587181	NZ_AMPN01000006.1 1..22395	NZ_KB890704.1 738253..783217	CADY01000068.1 1..34880
Bacteriocin	Unknown	415649..425903	740656..750895	701944..712183	NZ_AMPN01000269.1 2352..8553	NZ_KB890705.1 614326..624580	CADY01000124.1 50376..61254
Bacteriocin	Unknown	879961..891289	1167101..1178414	1125708..1137021	NZ_AMPN01000026.1 11838..21948	NZ_KB890709.1 5759..17087	CADY01000132.1 5648..13695
Lantipeptide	SapB	2713288..2735999	2912898..2935531	2877890..2900523	NZ_AMPN01000379.1 313..18317	NZ_KB890715.1 87381..116434	CADY01000176.1 1574..29018

*denotes genomic coordinates were edited manually

** denotes a gene cluster which was not annotated by antiSMASH 2.0

***denotes a gene cluster which is spread over multiple contigs (refer to S2 Table).

### Auxiliary biosynthetic capabilities of *Streptomyces albus*

Beyond the core metabolome, *S. albus* harbours 14 ‘auxiliary’ biosynthetic gene clusters. Auxiliary biosynthetic gene clusters are conserved to varying extents by *S. albus* strains, the details of which are summarized in Table 4. NRPS gene clusters were the most abundant class of biosynthetic system (7 out of 14 gene clusters) followed by hybrid NRPS / PKS systems (2 out of 14). As to be expected, the overwhelming majority of auxiliary gene clusters encode the production of unknown products (Table 4). Thus far, only one product of an auxiliary gene cluster has been elucidated, indigoidine. Indigoidine is a blue NRPS-derived pigment produced by *S. albus* J1074 and *S*. sp. LaPpAH-202. Interestingly, biosynthesis of indigoidine, at least in *S. albus* J1074 is repressed under normal laboratory growth conditions, and indigoidine production was only achieved by knocking-in the ermE* promoter upstream of core biosynthetic genes [30]. Although production of only one auxiliary metabolite has been analysed, bioinformatics analyses suggest that both *S*. sp. PVA 94-07 and *S*. sp. GBA 94-10 possess gene clusters coding for the biosynthesis of enterocin and a compound related to kijanamycin, which are both antibacterial agents [9].

**Table 4:** Auxiliary secondary metabolites produced by *Streptomyces albus*.

**Predicted biosynthetic system**	**Predicted product**	***S. albus* J1074**	***S*. sp. PVA-94-07**	***S*. sp. GBA 94-10**	***S*. sp. SM8**	***S*. sp. LaPpAH-202**	***S*. sp. S4**
Hybrid NRPS / PKS	Unknown	3011..61711	288401..349562	—	NZ_AMPN01000108.1 1..17827	NZ_KB890705.1 211618..272818	Multiple contigs**
NRPS	Unknown	1136316..1199422	—	—	—	Multiple contigs**	—
Lantipeptide	Unknown	2376688..2409159	2603848..2636308	2566778..2599136	—	NZ_KB890736.1 70394..102866	CADY01000169.1 12631..53654
NRPS	Unknown	3553726..3604015	—	—	Multiple contigs**	NZ_KB890716.1 353451..403740	Multiple contigs**
NRPS	Indigoidin	6336942..6381213	—	—	—	NZ_KB890733.1 244920..289191	—
Terpene	Unknown	—	274831..297419	284406..306988	NZ_AMPN01000169.1 36623..53502	NZ_KB890705.1 195282..217864	CADY01000116.1 145638..168220
Hybrid NRPS / PKS	Kijanimycin-like	—	397572..502620	366365..471413	—	—	—
Type I PKS	Unknown	—	771534..818373	731491..778330	—	—	—
NRPS	Unknown	—	3152743..3211020	3129401..3187816	—	—	—
NRPS	Unknown	—	3750249..3812487	3726395..3788633	—	—	—
NRPS	Unknown	—	4898187..4960375	4891468..4953657	—	—	—
NRPS*	Unknown		1..49621 (6957897..7007517)	2794..56173 (6969866..7023245)			
Type I PKS-butyrolactone*	Unknown		30737..134245 (6888273..6976781)	37289..140797 (6900242..6988750)			
Type II PKS	Enterocin	—	6259612..6302316	6251573..6294277	—	—	—

* Denotes a gene cluster harboured in duplicate; genomic coordinates for the additional copy are provide in braces.

**Denotes a gene cluster spread over multiple contigs, which are presented in S2 Table.

### Strain-specific metabolites produced by *Streptomyces albus*

In addition to core and auxiliary metabolites, *S. albus* strains harbour a total of 17 strain-specific gene clusters whose putative products comprise all of the major classes of secondary metabolites (Table 5). Each *S. albus* strain specifies at least one strain-specific gene cluster, which is consistent with *Salinispora arenicola, S. pacific* and *S. tropica* strains each encoding the production of ∼1.0 strain-specific polyketide or non-ribosomal peptide [7]. *S. sp.* PVA 94-07 and *S. sp.* GBA 94-10 harbour a single strain-specific gene cluster apiece, which is the fewest number specified out of all strains (Tables 2 and 5). However, eight gene clusters with unknown products are shared between *S. sp.* PVA 94-07 and *S. sp.* GBA 94-10 and are not harboured by other *S. albus* strains, suggesting that despite this, *S. sp.* PVA 94-07 and *S. sp.* GBA 94-10 produce a significant amount of novel chemistry. *S*. sp. S4 harbours six strain-specific gene clusters whose products represent 21% of its secondary metabolome, which is the most of any *S. albus* strain (Table 2) and may reflect its possible role as a defensive symbiont of fungus-growing ants [12]. Paulomycin, the product of a hybrid NRPS/PKS gene cluster encoded by *S. albus* J1074 is the only analysis of a strain-specific gene cluster thus far [30,43]. Although chemical analysis is required for confirmation, there is strong bioinformatics support to suggest that products of two of the strain-specific gene clusters encoded by *S. sp*. S4 are the hybrid type I / type III polyketide kendomycin and the type II polyketide fredericamycin [38]. The remaining 13 biosynthetic gene clusters harboured by *S. albus* strains are unknown. The antiSMASH 2.0-implementation of MultiGeneBlast [44] was used to identify the closest relative for each strain-specific gene cluster. Organisms harbouring putative orthologous gene clusters and the associated MultiGeneBlast score are reported in Table 5. A possible orthologue was identified for all but one strain-specific gene cluster specifying a bacteriocin harboured by *S. albus* S4 (Table 5).

**Table 5:** Strain-specific gene clusters encoded by *Streptomyces albus*.

**Predicted biosynthetic system**	**Predicted product**	**Coordinates**	**Closest relative (Accession, cumulative MultiGeneBlast Score)**
***S. albus* J1074**			
NRPS / Oligosaccharide	Paulomycin	684407..718548	*Streptomyces pristinaespiralis* ATCC 25486, NZ_CM000950.1, 3720
Bacteriocin	Unknown	2560714..2571226	*Streptomyces* sp. SPB74, NZ_GG770539.1, 683
***S*. sp. PVA-94-07**			
Lantipeptide	Unknown	1862908..1885125	*Streptomyces* sp. SPB74, NZ_GG770539.1, 4533
***S*. sp. GBA 94-10**			
Other*	Unknown	CM002272.1 609..41190	*Streptomyces* sp. W007, NZ_AGSW01000123.1, 3201
***S*. sp. SM8**			
Type II PKS	Unknown	NZ_AMPN01000020.1 1..23214	*Streptomyces pristinaespiralis* ATCC 25486, NZ_CM000950.1, 8748
Butyrolactone	Unknown	NZ_AMPN01000075.1 188..10692	*Streptomyces lavendulae*, AB434932.1, 1901
Bacteriocin	Unknown	NZ_AMPN01000145.1 80715..91067	*Streptomyces hygroscopicu* s, NZ_GG657754.1, 555
***S*. sp. LaPpAH-202**			
NRPS	Unknown	NZ_KB890705.1 132033..188388	*Streptomyces* sp. ATCC 700974, FN545130.1, 8976
NRPS	Unknown	NZ_KB890711.1 46489..98964	*Streptomyces hygroscopicus* ATCC 53653, NZ_GG657754.1, 6631
Type I PKS**	Unknown	NZ_KB890711.1 107544..132946 NZ_KB890725.1 1..5945 NZ_KB890733.1 334378..366455 NZ_KB890733.1 366455..396056	*Streptomyces tsukubaensis* NRRL18488, AJSZ01000000, ***
***S*. sp. S4**			
Other	Unknown	CADY01000053.1 1..9842	*Frankia* sp. EUN1f, NZ_ADGX01000038.1, 551
Type I PKS / Type III PKS	Kendomycin	CADY01000062.1 1..35064	*Streptomyces griseus* XylebKG-1, NZ_GL877172.1, 4372
NRPS	Mannopeptimycin-like	CADY01000178.1 54079..109040	*Streptomyces venezuelae* ATCC 10712, FR845719.1, 5568
Butyrolactone	Unknown	CADY01000186.1 1..29569	*Streptomyces* sp. W007, NZ_AGSW01000147.1, 1300
Bacteriocin	Unknown	CADY01000195.1 1..2452	—
Type II PKS	Fredericamycin	CADY01000200.1 57212..86518	*Streptomyces griseus*, AF525490.2, 11189

*Denotes a gene cluster encoded on a plasmid

**Denotes a gene cluster composed of partial antiSMASH gene clusters that likely represent a single cluster according to NaPDoS analysis

***AntiSMASH did not report a cumulative MultiGeneBlast score, because the gene cluster is spread over multiple contigs

### Conclusions and perspectives

The genomes of *S. albus* isolates have been sequenced more than any other species of *Streptomyces*. The putative biosynthetic capabilities of six *S. albus* strains were analysed here, which identified a core secondary metabolome specified by 18 biosynthetic gene clusters as well as 14 auxiliary gene clusters and 16 strain-specific gene clusters. The products of 29 of the 48 gene clusters identified in this analysis are unknown, representing an attractive reservoir of compounds that may have useful medicinal or industrial applications or may otherwise comprise a chemically interesting scaffold. The flurry of recent analyses investigating secondary metabolism of *S. albus* strains have collectively resulted in assigning products to 15 of the 25 gene clusters encoded by *S. albus* J1074, rivaling what is known about *S. coelicolor* which has been rigorously studied for over half a century [45]. Robust and thorough bioinformatics approaches that prioritise taxonomic uniqueness of producing organisms and novel gene clusters will drive the discovery of new compounds. However, many of the gene clusters encoded by streptomycetes are not expressed under normal laboratory growth conditions. In order to therefore maximally exploit the biosynthetic potential of these organisms the regulation of biosynthetic systems must be refactored in the native host or cloned and heterologously expressed variants whose expression has been engineered. These efforts are aided by recent advances in the selective cloning of large genomic DNA inserts [46,47] and will be further aided by the decreasing price of custom DNA synthesis and the ability to assemble these fragments in yeast [48].

## Materials and Methods

### Phylogenetic analyses

The Genomic Blast service hosted by NCBI was used to query all complete and draft genomic sequences from bacteria taxonomically classified as *Streptomyces* spp. (taxid = 1883) with partial DNA sequences for *atpD, gyrA, recA, rpoB* and *trpB*, which corresponded to the sequences targeted by oligonucleotide primers used by [26,27] to infer a multilocus phylogeny. FASTA sequence files for relevant accession numbers were downloaded from Genbank using Batch Entrez and BedTools 2.19.0 [49] was used to extract nucleotide sequence ranges reported in the blast search into a multifasta file. The BioPerl [50] script shortenID.pl (http://nebc.nox.ac.uk/scripts/parse/shortenID.pl ) written by Bela Tiwari, NERC Environmental Bioinformatics Centre,was used to shorten headers for FASTA entries and the BioPerl script split_multifasta.pl (http://iubio.bio.indiana.edu/gmod/genogrid/scripts/split_multifasta.pl ) written by the Genome Informatics Lab at Indiana University was used to generate individual FASTA files from the resulting multifasta output from BedTools. DNA sequences were aligned using eight iterations of the MEGA 5.2.2 implementation of Muscle [51] and were trimmed to the same length (including gaps) and subsequently concatenated in the order: *aptD-gyrB-recA-rpoB-trpB*. Phylogenetic relationships were inferred from the concatenated sequences by approximate maximum likelihood analysis using FastTree 2.1.7 [52]. *Mycobacterium tuberculosis* H37Rv was used as an outgroup and MEGA 5.2.2 was used to visualise and edit the tree. Concatenated *aptD-gyrB-recA-rpoB-trpB* sequences were grouped into operational taxonomic units (OTUs) using the MacQiime v1.80 implementation of UCLUST [28,53] with a shared identity threshold of 97%.

### Analysis of secondary metabolite gene clusters

**Figure 2:**
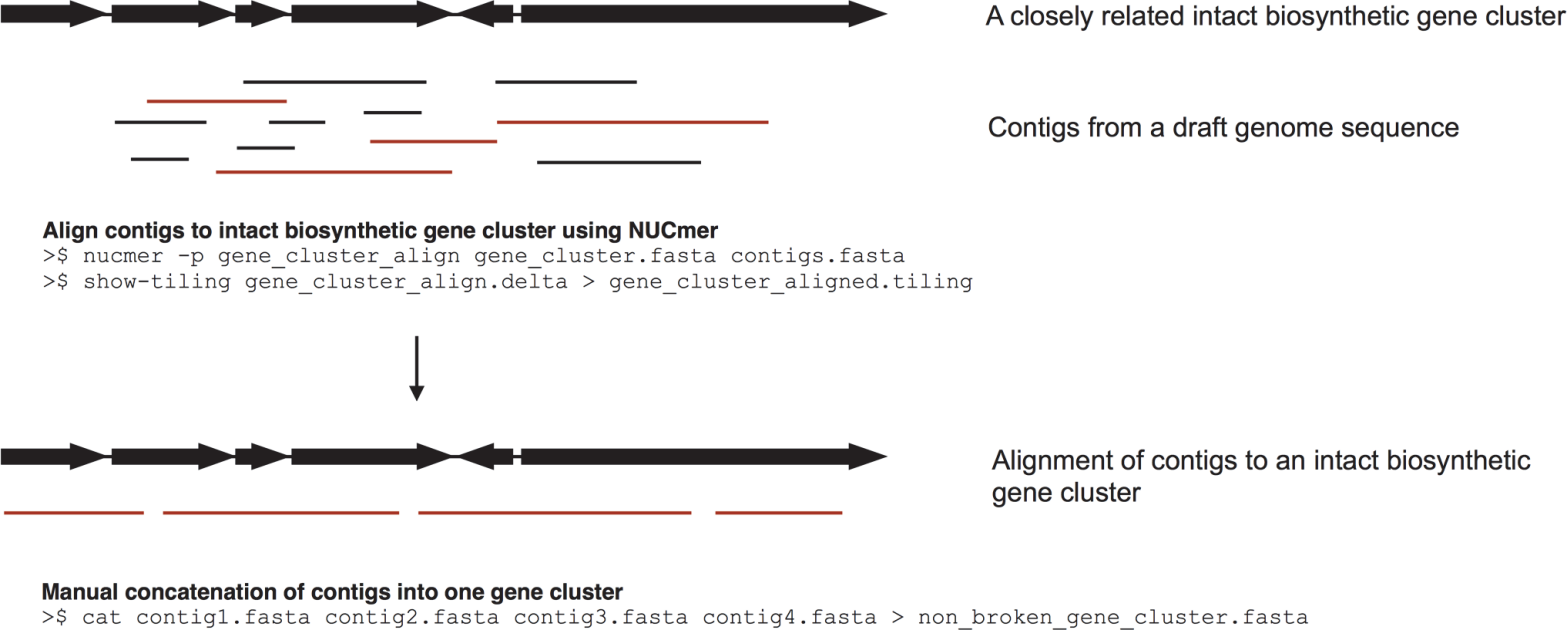
Diagrammatic workflow of the NUCmer approach used to piece together a biosynthetic gene clusters spread over more than one contig. NUCmer is part of the MUMmer [55] and can be downloaded from http://sourceforge.net/projects/mummer/. NUCmer will align contigs from draft genomes to an intact gene cluster with high shared nucleotide identity. Commands used to perform an analysis of this type are given. Black arrows represent a biosynthetic gene cluster; black and red lines represent contigs in a draft genome sequence.

Genome sequences analysed here were downloaded from Genbank or EMBL (see Table 1 for accessions) and putative biosynthetic gene clusters for secondary metabolites were identified using the default settings in the web implementation of antiSMASH 2.0 [29] and the nucleotide sequence for each gene cluster was extracted from the outputted Genbank files using EMBOSS utility seqret [54]. The large number of contigs in some draft genomes caused antiSMASH 2.0 to identify numerous broken or incomplete gene clusters. This was a particular problem with polyketide synthase gene clusters. In order to minimise the impact of broken gene clusters on this analysis, the gene clusters identified from the fully sequenced genome of *S. albus* J1074 were used as a reference for NUCmer [55] alignments of gene clusters from draft genome sequences. A diagrammatic workflow of this approach is displayed in Fig. 2. Gene clusters from draft genomes that aligned to the same *S. albus* J1074 gene cluster were subsequently concatenated into a single FASTA file and considered a single gene cluster. A For gene clusters in which *S. albus* J1074 did not harbour a homologous cluster, NaPDoS [56] was used to identify and extract ketosynthase domains from gene clusters identified by antiSMASH 2.0. The resulting amino acid sequences were aligned by the Geneious 7.1.5 implementation of Muscle (eight iterations) and a neighbour-joining phylogenetic tree was inferred from the alignment using the Geneious 7.1.5 tree builder with a Jukes-Cantor distance model (not shown). A customised blast database was generated using Blast 2.2.29+ [57] and a combination of blast analysis and whole gene cluster alignments using Mauve 2.3.1 [58] were used to both further refine broken gene clusters in draft genome sequences and to ascertain the conservation of secondary metabolite gene clusters across the *S. albus* clade. Self vs. self blastn analyses were used to identify and remove duplicate gene clusters.

## Supporting Information

S1 Table Table of operational taxonomic units (97% shared identity) of concatenated *aptD-gyrB-recA-rpoB-trpB* sequences from 120 sequenced streptomycetes and *M. tuberculosis*. (PDF)

S2 Table Table of genomic details for gene clusters encoded over multiple contigs. (PDF)
