# Supplementary material for: Strain-Level Diversity of Secondary Metabolism in Streptomyces albus
Source: PLoS One. 2015 Jan 30;10(1):e0116457. doi: 10.1371/journal.pone.0116457 (PMC4312078; doi:10.1371/journal.pone.0116457)
Supplement: S1 Table — (PDF) [file pone.0116457.s001.pdf]

OTU ID	Organisms						
1	*M. tuberculosis* H37Rv						
2	*S. acidiscabies* 84104						
3	*S. afghaniensis* 772	*S. chartreusis* NRRL 12338					
4	*S. albulus* CCRC 11814	*S. albulus* PD1					
5	*S. albus* J1074	*S. sp.* PVA 9407	*S*. sp. GBA 9410	*S*. sp. PPC42	*S*. sp. SM8	*S*. sp. LaPpAH202	*S*. sp. S4
6	*S. aurantiacus* JA 4570 Seq90						
7	*S. auratus* AGR0001						
8	*S. avermitilis* MA4680						
9	*S. bingchenggensis* BCW-1						
10	*S. cattleya* NRRL 8057						
11	*S. clavuligerus* ATCC 27064						
12	*S. coelicoflavus* ZG0656	*S. lividans* 1326	*S. coelicolor* A3(2)	*S*. sp. FXJ7.023			
13	*S. collinus* Tu 365						
14	*S. davawensis* JCM 4913						
15	*S. exfoliatus* DSM 41693						
16	*S. flavidovirens* DSM 40150						
17	*S. flavogriseus* ATCC 33331	*S*. sp. PAMC26508					
18	*S. ghanaensis* ATCC 14672	*S. viridosporus* T7A					
19	*S. griseoaurantiacus* M045						
20	*S. griseoflavus* Tu4000						
21	*S. griseus* subsp. *griseus* NBRC 13350	*S. griseus* XylebKG1	*S*. sp. W007				
22	*S. himastatinicus* ATCC 53653						
23	*S. hygroscopicus* subsp. *jinggangensis* 5008	S. *hygroscopicus* subsp. *jinggangensis* TL01					
24	*S. ipomoeae* 9103						
25	*S. mobaraensis* NBRC 13819						
26	*S. pristinaespiralis* ATCC 25486						
27	*S. prunicolor* NBRC 13075						
28	*S. purpureus* KA281						
29	*S. rapamycinicus* NRRL 5491						
30	*S. rimosus* subsp. *rimosus* ATCC 10970						
31	*S. roseochromogenes* subsp. oscitans						
32	*S. roseosporus* NRRL 15998	*S. roseosporus* NRRL 11379	*S. globisporus* C1027	S. sp. HCCB10043			
33	*S. scabies* 87.22	*S. bottropensis* ATCC 25435					
34	*S. somaliensis* DSM 40738						
35	*S*. sp. 142MFCol3.1						
36	*S*. sp. 303MFCol5.2						
37	S. sp. 351MFTsu5.1						
38	*S*. sp. AA0539						
39	*S*. sp. AA1529						
40	*S*. sp. ATexABD23						
41	*S*. sp. BoleA5						
42	*S*. sp. CNB091						
43	*S*. sp. CNH099						
44	*S*. sp. CNH189						
45	*S*. sp. CNQ329	*S*. sp. CNT371	*S*. sp. CNQ865				
46	*S*. sp. CNR698	*S*. sp. CNS615	*S*. sp. CNT302				
47	*S*. sp. CNS335						
48	*S*. sp. CNT360						
49	*S*. sp. CNT372						
50	*S*. sp. CNY228						
51	*S*. sp. CNY243						
52	*S*. sp. DpondAAB6						
53	*S*. sp. DvalAA83	*S*. sp. SirexAA-E					
54	*S*. sp. e14	*S*. sp. Amel2xE9					
55	*S*. sp. FxanaC1						
56	*S*. sp. FxanaD5	*S. gancidicus* BKS 1315					
57	*S*. sp. HGB0020						
58	*S*. sp. HmicA12						
59	*S*. sp. HPH0547						
60	*S*. sp. KhCrAH340	*S*. sp. KhCrAH244	*S*. sp. KhCrAH337	*S*. sp. KhCrAH40	*S*. sp. LaPpAH95	*S*. sp. LaPpAH165	
61	*S*. sp. LaPpAH108						
62	*S*. sp. Mg1						
63	*S*. sp. MspMPM5						
64	*S*. sp. PsTaAH124						
65	*S*. sp. SA3 actF	*S*. sp. SPB78	*S*. sp. Tu6071				
66	*S*. sp. ScaeMPe10	*S. fulvissimus* DSM 40593	*S*. sp. Wigar10	*S*. sp. CcalMP8W			
67	*S*. sp. SPB74						
68	*S*. sp. SS						
69	*S*. sp. TAA486						
70	*S*. sp. TOR3209						
71	*S. sulphureus* DSM 40104						
72	*S. sulphureus* L180						
73	*S. sviceus* ATCC 29083	*S. canus* 299MFChir4.1					
74	*S. thermolilacinus* SPC6						
75	S. tsukubaensis NRRL18488						
76	*S. turgidiscabies* Car8						
77	*S. venezuelae* ATCC 10712						
78	*S. violaceusniger* Tü 4113						
79	*S. viridochromogenes* DSM 40736						
80	*S. viridochromogenes* Tü 57						
81	*S. vitaminophilus* DSM 41686						
82	*S. xinghaiensis* S187						
83	*S. zinciresistens* K42						
