# Supplementary material for: Strain-Level Diversity of Secondary Metabolism in Streptomyces albus
Source: PLoS One. 2015 Jan 30;10(1):e0116457. doi: 10.1371/journal.pone.0116457 (PMC4312078; doi:10.1371/journal.pone.0116457)
Supplement: S2 Table — (PDF) [file pone.0116457.s002.pdf]

Predicted biosynthetic system	Predicted product	*S.* albus J1074 coordinates	*S.* sp. SM8	*S.* sp. LaPpAH-202	*S.* sp. S4
NRPS	Gramicidin-like	3877105..3982798			
			NZ_AMPN01000069.1	NZ_KB890724.1	
			NZ_AMPN01000380.1	NZ_KB890722.1	
			NZ_AMPN01000067.1		
			NZ_AMPN01000316.1		
			NZ_AMPN01000031.1		
Type I PKS	Candicidin	6566408..6721648			
			NZ_AMPN01000181.1	NZ_KB890733.1	CADY01000095.1
			NZ_AMPN01000212.1	NZ_KB890720.1	CADY01000094.1
			NZ_AMPN01000210.1	NZ_KB890721.1	CADY01000093.1
			NZ_AMPN01000209.1	NZ_KB890728.1	CADY01000092.1
			NZ_AMPN01000314.1	NZ_KB890710.1	CADY01000091.1
			NZ_AMPN01000380.1		
			NZ_AMPN01000177.1		
			NZ_AMPN01000337.1		
			NZ_AMPN01000338.1		
			NZ_AMPN01000339.1		
Hybrid NRPS / PKS	Antimycin	6730563..6755198			
			NZ_AMPN01000050.1		
			NZ_AMPN01000430.1		
			NZ_AMPN01000462.1		
			NZ_AMPN01000393.1		
NRPS	Unknown	6755272..6776675			
			NZ_AMPN01000393.1		
			NZ_AMPN01000411.1		
			NZ_AMPN01000174.1		
NRPS-independent siderophore synthase	Aerobactin-like	1268164..1283196			
			NZ_AMPN01000222.1		
			NZ_AMPN01000095.1		
Hopene / Squalene synthase	Hopanoids	308626..335220			
			NZ_AMPN01000054.1		
			NZ_AMPN01000311.1		
NRPS	Unknown	3553726..3604015			
			NZ_AMPN01000145.1		CADY01000201.1
			NZ_AMPN01000217.1		CADY01000202.1
NRPS	Unknown	1136316..1199422			
				NZ_KB890708.1	
				NZ_KB890714.1	
Hybrid NRPS / PKS	Unknown	3011..61711			
					CADY01000116.1
					CADY01000117.1
Type I PKS	Unknown	6776680..6838639			
			NZ_AMPN01000106.1		CADY01000087.1
			NZ_AMPN01000245.1		CADY01000089.1
